# The Role of MicroRNA and Microbiota in Depression and Anxiety

**DOI:** 10.3389/fnbeh.2022.828258

**Published:** 2022-03-01

**Authors:** Julia M. Rosa, Douglas A. Formolo, Jiasui Yu, Thomas H. Lee, Suk-yu Yau

**Affiliations:** ^1^Department of Rehabilitation Sciences, Hong Kong Polytechnic University, Hung Hom, Hong Kong SAR, China; ^2^Mental Health Research Center (MHRC), Hong Kong Polytechnic University, Hung Hom, Hong Kong SAR, China; ^3^Research Institute for Smart Aging (RISA), Hong Kong Polytechnic University, Hung Hom, Hong Kong SAR, China; ^4^Neurocentre Magendie, INSERM U1215, University of Bordeaux, Bordeaux, France

**Keywords:** microRNA, depression, anxiety, microbiota, psychiatric disorders

## Abstract

Depression and anxiety are devastating disorders. Understanding the mechanisms that underlie the development of depression and anxiety can provide new hints on novel treatments and preventive strategies. Here, we summarize the latest findings reporting the novel roles of gut microbiota and microRNAs (miRNAs) in the pathophysiology of depression and anxiety. The crosstalk between gut microbiota and the brain has been reported to contribute to these pathologies. It is currently known that some miRNAs can regulate bacterial growth and gene transcription while also modulate the gut microbiota composition, suggesting the importance of miRNAs in gut and brain health. Treatment and prevention strategies for neuropsychiatric diseases, such as physical exercise, diet, and probiotics, can modulate the gut microbiota composition and miRNAs expressions. Nonetheless, there are critical questions to be addressed to understand further the mechanisms involved in the interaction between the gut microbiota and miRNAs in the brain. This review summarizes the recent findings of the potential roles of microbiota and miRNA on the neuropathology of depression and anxiety, and its potential as treatment strategies.

## Introduction

MicroRNAs (miRNAs) are single-stranded non-coding RNAs, with an average of 22 nucleotides in length, which function as the posttranscriptional regulators of gene expression, primarily through translational repression (Ha and Kim, 2014). Most miRNAs are transcribed from DNA sequences into the primary miRNAs, then processed into the precursor miRNAs, and finally the mature miRNAs (O’Brien et al., 2018). miRNAs are involved in regulating numerous developmental and physiological processes (Ha and Kim, 2014). They are also secreted into the extracellular fluid and serve as the signaling molecules to facilitate cell-to-cell communication (Sohel, 2016). The aberrant expressions of miRNAs are associated with the pathogeneses of cancer (Cui et al., 2019), aging (Kinser and Pincus, 2020), and neuropsychiatric disorders (Xu et al., 2012). Hence, the expressions of miRNAs could be the biomarkers for these diseases, and specifically, miRNAs are suggested as the new pharmacological targets and biomarkers for treating and diagnosing depression and anxiety (Scott et al., 2015; Yuan et al., 2018).

The composition of gut microbiota, on the other hand, is another factor that is associated with psychiatric disorders. Gut microbiota is the microbial flora that inhabits the intestine and is involved in digestion, including bowel movement, food digestion, as well as water and nutrient absorption (Grochowska et al., 2018). Interestingly, changes in the gut microbiota composition may eventually contribute to the pathogeneses of neuropsychiatric disorders. Patients with depression, for instance, have altered intestinal microbiota compositions (Amirkhanzadeh Barandouzi et al., 2020). In addition, changes in the microbiota due to antibiotics administration and other conditions can induce depression-like behavior in animals. Chronic unpredictable mild stress promotes anxiety and depression-like behaviors in mice associated with an altered gut microbiota profile, whereas mice colonized with gut microbiota from stressed animals show similar behaviors (Li et al., 2019). Likewise, fecal microbiota transplantation from depressed patients to microbiota-depleted rats can induce depression and anxiety-like behaviors (Kelly et al., 2016).

The miRNA expressions in the brain and the gut microbiota composition are associated with the pathogenesis of depression and anxiety. Critically, miRNAs secreted in the circulation can modulate the microbiota composition, whereas the microbiota composition influences the miRNA expressions in limbic structures that are important for mood regulation. In-depth reviews of the roles of miRNAs and the microbiome in psychiatric disorders such as anxiety and depression can be referred to Meydan et al. (2016), Allen and Dwivedi (2020), Huang and Wu (2021), and Simpson et al. (2021). Here, we review the latest literature by discussing the potential gut microbiota-miRNA crosstalk in modulating depression and anxiety-related disorders, highlighting the relevance of miRNA as the new biomarkers and potential intervention strategies.

## The Roles of Microbiota on Depression, Anxiety, and MicroRNA Expression

### The Potential Role of Microbiota in Depression

Depression is characterized by sad or irritable mood accompanied by autonomic and cognitive changes that substantially affect the individual’s functionality (American Psychiatric Association, 2013). Depression can be understood from a neurochemical perspective, where a downregulation in the monoaminergic transmission associated with dorsal raphe nucleus (DRN) activity results in poor mood regulation (Coppen, 1967; Cosci and Chouinard, 2019). On the other hand, as it has been more recently proposed, depression is recently proposed as a result from the disintegration of systems and mal-functioning circuits (Manji et al., 2003; Castrén, 2013; Marsden, 2013). In agreement with that, it was recently found that patients with major depressive disorder present lower synaptic density in the dorsolateral prefrontal cortex (PFC), anterior cingulate cortex, and hippocampus (Holmes et al., 2019), all these structures that are involved with emotional reappraisal (Buhle et al., 2014), sadness (Drevets et al., 1997), and cognition (Anacker and Hen, 2017). Increasing structural and synaptic plasticity within these systems have been proposed to be linked to antidepressant actions (Duman et al., 2016).

The relationship between microbiota composition and depression has long been established. Depressed patients have altered microbiota composition compared with healthy controls and decreased microbiota diversity and richness (Kelly et al., 2016; Zheng et al., 2016). Moreover, fecal transplantation from depressed patients to germ-free (GF) rodents induces depressive phenotype that is usually followed by increased anxiety-like behaviors (Kelly et al., 2016; Zheng et al., 2016). However, the mechanisms by which the microbiota affects the brain remain poorly understood. The inflammatory system has been suggested as a possible pathway among the candidates due to its relevance for neuronal development and plasticity (Fung et al., 2017). Indeed, a recent meta-analysis has confirmed that several psychiatric disorders, including depression and anxiety, present reduced gut bacteria responsible for producing the anti-inflammatory butyrate along with increased pro-inflammatory generating bacteria (Nikolova et al., 2021). The enteric nervous system also represents a pathway by which alterations in the gut microbiota can directly inform the central nervous system (Bravo et al., 2011). Severing the vagal nerve can block some microbiota effects over central signaling systems (Bravo et al., 2011). Finally, the microbiota composition has been shown to modulate the individuals’ response to stress to the same extent to which chronic stress can shape the microbiota composition (Foster and McVey Neufeld, 2013).

In the learned helplessness paradigm, vulnerable rats have altered microbiota composition compared with resilient and control rats, suggesting the microbiome influences stress susceptibility (Zhang et al., 2019). On the other hand, early-life stress induced by maternal separation induces dysbiosis in the adult offspring displaying depressive phenotype (O’Mahony et al., 2009; de Palma et al., 2015). Likewise, chronic probiotic treatment significantly improves depressive phenotype induced by maternal separation in the adult offspring (Desbonnet et al., 2010). Of note, the beneficial effects of probiotic administration are independent to dysbiosis, such that chronic treatment with a *Lactobacillus* strain reduces anxiety- and depression-like behavior in the physiological condition and reduces corticosterone reactivity in response to acute stress (Bravo et al., 2011). Moreover, both acute (ketamine) (Yang et al., 2017) and chronic (fluoxetine) (Zhang et al., 2021) antidepressant treatment are associated with an increased relative abundance of the gut microbiota. Therefore, stress directly influences the microbiota composition, and the microbiota *per se* can impact stress resilience.

Microbiota composition can directly affect the central nervous systems associated with the glucocorticoid response. Depleted microbiota is associated with increased hippocampal noradrenaline and reduced serotonin levels (de Palma et al., 2015; Hoban et al., 2016), increased neuronal activation in areas associated with stress response, such as the paraventricular nucleus of the hypothalamus (PVN) and bed nucleus of the stria terminalis (BNST) (Wu et al., 2021), and decreased gene expression of glucocorticoid receptors and CHF in the hippocampus and amygdala (Hoban et al., 2016). Likewise, mice with depleted microbiota have increased basal corticosterone levels and HPA reactivity in response to acute stress, which can be reversed by microbiota recolonization (Clarke et al., 2013; Wu et al., 2021). Moreover, chronic probiotic treatment reduces the corticosterone reactivity to acute stress and improves depressive phenotype (Bravo et al., 2011; Liang et al., 2015). Noteworthy, probiotic antidepressant effects are associated with improved expression of GABA receptors (Bravo et al., 2011) and restored BDNF, noradrenaline, and serotonin levels in the hippocampus and mPFC (Liang et al., 2015; Sun et al., 2018). Moreover, such probiotic effects are abolished upon vagotomy (Bravo et al., 2011), suggesting the enteric nervous system as a direct communication pathway in the gut-brain axis.

Remarkably, GF rodents tend to present reduced anxiety- and depression-like behaviors (Heijtz et al., 2011; Clarke et al., 2013; de Palma et al., 2015; Wu et al., 2021) and are resilient to behavioral impairments induced by maternal separation (de Palma et al., 2015). Such aberrant profile is associated with reduced hippocampal BDNF expression, increased hippocampal and striatal serotonin levels, and plasma tryptophan concentrations (Heijtz et al., 2011; Clarke et al., 2013). Notably, microbiota recolonization in adult life fails to restore some features associated with early life microbiota deficiency, mainly concerning the aberrant hippocampal monoaminergic system activity (Clarke et al., 2013), suggesting a long-lasting effect of early life dysbiosis. Unexpectedly, GF mice that are resilient to maternal separation display depressive phenotypes upon microbiota colonization in adult life (de Palma et al., 2015), suggesting changes of microbiota profile in adulthood can affect stress resilience.

### The Potential Roles of Microbiota in Anxiety

Clinical anxiety is characterized by a broad range of exaggerated and enduring symptoms, such as increased arousal and excessive fear and anxiety-related responses, including autonomic changes, such as increased heart rate and sweating, often in the absence of any real threat or danger (Hoffman and Mathew, 2008). The mechanisms leading to anxiety pathophysiology are not yet fully elucidated, although studies have indicated that anxiety disorders are multifactorial, possibly involving gene and environmental interaction (Hoffman and Mathew, 2008). Similar to depression, ample evidence has shown that the gut microbiota plays a major role in anxiety pathogenesis (Nishino et al., 2013; Crumeyrolle-Arias et al., 2014; de Palma et al., 2015).

Several early studies have shown anxiolytic-like behaviors in GF rodents, as indicated by increased exploration of unfamiliar areas in the open-field test (OFT) and elevated plus-maze (EPM) test (Heijtz et al., 2011; Crumeyrolle-Arias et al., 2014). A further study has demonstrated the infectious effect of microbiota on anxiety by colonizing GF adult mice with microbiota from specific pathogen-free (SPF) mice (Neufeld et al., 2011). This effect returns to normal as infant rodents enter adulthood (Nishino et al., 2013). Also, offspring subjected to microbiota colonization has shown a significant reduction in spontaneous locomotor activity and anxiety-like behavior (Heijtz et al., 2011).

Anxiety-like behavior can be caused by a dysbiosis of the gut microbiota (Carding et al., 2015; Kc et al., 2020). Rodent studies have demonstrated that intestinal microbiota disruption induced by stress (Bharwani et al., 2017), or high-fat diet (Bruce-Keller et al., 2017), or antibiotics (Park et al., 2021) leads to anxiety-like behavior, which can be restored to normal by probiotic administration, including *Escherichia coli* (Park et al., 2021) and *B. longum* (Pinto-Sanchez et al., 2017). These results suggest that anxiety may arise from perturbations in the gut microbiota caused by external conditions. However, the precise mechanisms whereby the dysbiosis of microbiota affects anxiety-like behaviors is still unclear. Recent findings show that disrupted gut microbiota is associated with autoimmune disease and neuroinflammation, as indicated by increased serum levels of the pro-inflammatory cytokines IL-6 and NF-κB (Estes and McAllister, 2015; Schnorr and Bachner, 2016), suggesting that inflammatory response could be potential mediator of gut dysbiosis on inducing anxiety.

The Fusobacterium, such as *Lachnospiraceae* (Luna and Foster, 2015) and *Ruminococcaceae* (Tengeler et al., 2020) increases levels of pro-inflammatory cytokine in association with social avoidance. Studies have shown that the microbiota induces release of cytokines and associated pro-inflammatory proteins into the blood (such as TNF-α and interferon-γ), which in turn impair epithelial function and intestinal permeability (Lee and Lee, 2014; Martin-Subero et al., 2016), and activate the intestinal immune cells (de Palma et al., 2017) and primary afferent nerves (Burton and Gebhart, 1995). Anxiety and depression are commonly associated with dysregulation of the HPA axis (van den Bergh et al., 2008), whereas the gut microbiota has been shown essential for regulating the HPA axis activity (Messaoudi et al., 2011; Xu et al., 2020). Dysbiosis-induced release of cytokines may contribute to anxiety via modulation of the HPA axis (Martin-Subero et al., 2016).

Previous studies have found that GF mice are more susceptible to stress-induced hyperactivation of the HPA axis resulting in elevated levels of adrenocorticotropic hormones (ACTH), corticotropin-releasing hormone (CRH), and corticosterone (Huo et al., 2017; Frankiensztajn et al., 2020). However, HPA axis hyperactivity returns to normal after colonization with commensal bacteria (Sudo et al., 2004). Furthermore, rats subjected to maternal separation (an animal model of early life adversity) showed dysbiosis depending on activation of the HPA axis (Cong et al., 2016). However, probiotic (*Lactobacillus spp.*) treatment normalizes basal cortisol levels and prevents hyperactivation of the HPA axis in offspring from dams with microbiota depletion (Bailey and Coe, 1999).

Lipopolysaccharide (LPS) and peptidoglycan also play critical roles in activating the HPA axis. Several studies have shown that stress not only simulates NF-κB activation and monocyte migration to the intestine (Jang et al., 2018) but also improves the density of *Aspergillus* and *E. coli* in the gut microbiota and increases fecal and serum LPS levels in rodents with anxiety-like behavior (Frankiensztajn et al., 2020). Further investigation suggests that intraperitoneally injection with LPS triggers persistent depression-like behavior in adolescent female mice and anxiety in adult male mice (Yahfoufi et al., 2021). Conversely, pubertal probiotic administration prevents LPS-induced depression/anxiety-like behaviors (Sylvia et al., 2018; Yahfoufi et al., 2021). These results support the hypothesis that LPS from proteobacteria causes an inflammatory response in the gastrointestinal tract, which then activates NF-κB signaling and thus the HPA axis, and consequently leads to anxiety (Girard-Joyal and Ismail, 2017; Yahfoufi et al., 2021).

Gut-brain communication has been demonstrated to transmit via the vagal nerve (Bercik et al., 2011; Breit et al., 2018; Fülling et al., 2019). Several studies have shown that the gut microbiota may communicate with the brain via intermediate intestinal cells and dendritic cells, conveying endocrine and neurological signals (Westfall et al., 2017; Huh and Veiga-Fernandes, 2020). In addition, microbial metabolites are potential regulators of neurotransmitter synthesis, for instance short-chain fatty acids, trypsin, 5-hydroxytryptamine, glutamate, and dopamine (Dalile et al., 2019). Thus, there are implications that microbial metabolites facilitate gut-brain communication and behavior regulation. The vagal nerve is chemosensitive and regulates anxiety-like behavior via orexigenic and anorexigenic neuropeptides secreted by enteroendocrine cells (Forsythe et al., 2014). Additionally, the vagal nerve responds to multiple signaling factors released by mast cells and lymphoid cells, including 5-HT and CRH, suggesting the enteric nervous system is also involved in modulating the HPA axis (Gui, 1998).

Anatomical studies have also shown that the sensory neurons of the submucous plexus are in contact with the microbiota and are involved in synaptic neogenesis with the motoneurons of the intestine (Furness et al., 2014), which is associated with the regulation of anxiety-like behaviors (Burokas et al., 2015). Moreover, *Campylobacter jejuni* increases the expression of c-Fos in vagal afferent brain regions and induces anxiety in mice (Goehler et al., 2005). Additionally, probiotics, such as *B. longum*, can relieve anxiety through enteric modulation in mice with colitis (Khoshdel et al., 2013).

Probiotics and prebiotics, which can promote the balance of gut microbiota, are of great interest as they also promote anxiety relief (Cryan and O’Mahony, 2011). Decreased anxiety-like behavior and plasma corticosterone are observed after prebiotic treatment (fructo-oligosaccharides) in stressed mice (Burokas et al., 2017). Such prebiotic strain also improves the mRNA expression of γ-aminobutyric acid (GABA) receptors in the hippocampus (Burokas et al., 2017). GABA is one of the major inhibitory neurotransmitters and can be synthesized by intestinal *Lactobacilli* and *Bifidobacteria* (Barrett et al., 2012). In addition, dietary probiotics have been shown to support gastrointestinal remodeling by increasing circulating glutathione and reducing inflammatory markers (Aslam et al., 2020).

In summary, the neurobiological study of anxiety and gut-CNS connections has revealed potential ways in which microbial disturbances can result in mood and behavior alterations. Although animal experiments have confirmed the use of probiotics to treat anxiety and depression, further mechanistic studies and clinical trials are required to provide scientific evidence on their clinical use.

### Effects of Microbiota on Modulating MicroRNA Expression

Recent studies show that microbiota depleted animals have altered expression of miRNA levels in addition to anxiety-like behavior (Hoban et al., 2017), supporting the role of microbiota in influencing the levels of miRNAs. Absence of microbiota in adolescent GF mice results in dysregulation of transcriptome expression in the hippocampus and reduced anxiety-like behavior (Liu et al., 2020). In another study, GF and antibiotic-induced depletion of the microbiota changes the miRNA expression in the amygdala and PFC (Hoban et al., 2017; Table 1). In the amygdala, miR-183-5p and miR-182-5p levels are decreased and subsequently normalized by colonization (Hoban et al., 2017). Both miRNAs are linked with amygdala-dependent stress- and fear-related behaviors (Bocchio-Chiavetto et al., 2013; Griggs et al., 2013). Moreover, miR-219a-2-3p expression in the same brain regions is altered in GF and antibiotic-treated mice (Hoban et al., 2017). Moreover, evidence shows that miR-219a-2-3p/miR-219-3p are altered in the basolateral amygdala following social defeat stress (Chen et al., 2015).

**TABLE 1:** Microbiota-induced modulation of miRNA levels in brain regions related to depression/anxiety.

	Microbiota depletion strategy	Altered miRNAs compared to control	Behavioral alterations	Results after microbiota colonization	References
Male Swiss Webster	Germ free	↑ miR-3535, miR-187-3p, miR-369-5p (amygdala) ↓ miR-182-5p, miR-183-5p, 219a-2-3p (amygdala) ↑ miR-219a-2-3p (PFC)	–	Restored: miR-182-5p, miR-182-3p, 219a-2-3p, miR-122-5p	Hoban et al., 2017
Male Sprague Dawley	Cocktail of antibiotics	↑ miR-369-3p (amygdala) ↓ miR-206-3p, 219a-2-3p (amygdala) ↓ 219a-2-3p (PFC)	–	–	Hoban et al., 2017
Male C57BL/6J mice	Germ free	↑ miR-184-3p, miR-344c-3p, miR-92b-5p, miR-342-5p, miR-380-3p, miR-760-3p, miR-485-3p (amygdala) ↓ miR-874-3p, miR-204-5p, miR-211-5p, miR-1298-5p, miR-448-3p (amygdala)	↓ Fear memory	Restored: behavior	Hoban et al., 2018
Male Balb/c mice	Germ free	↑ miR-190a-5p, miR-539-5p (hippocampus) ↓ miR-3095-3p, miR-363-5p, miR-421-3p, miR-673-3p, miR-758-5p (hippocampus)	↓ Anxiety-like behavior	No changes in behavior Restored: All microRNAs	Chen et al., 2017

*Altered microRNAs in microbiota depletion strategies in brain regions involved with depression and anxiety. ↓ (Decrease levels) and ↑ (Increase levels).*

The microbiome regulates amygdala-dependent fear and anxiety circuitry, and miRNAs are suggested as key mediators (Scott et al., 2015). GF mice have impaired auditory fear conditioning (Scott et al., 2015), which can be related to the apparent incapacity of GF mice to retain the association between conditioned (tone) and unconditioned (shock) stimuli when compared with controls (Hoban et al., 2018). After fear conditioning, miR-34b-5p, miR-34c-5p, and miR-34b-3p are downregulated (Hoban et al., 2018). Interestingly, it is shown that deletion of this miRNA family is related to anxiety resilience in stressed mice and reduced fear memory (Andolina et al., 2016). Together, these data indicate that microbiota and miRNA can be targeted for treating fear- and anxiety-related disorders.

Levels of seven miRNA, including miR-190a-5p, miR-3095-3p, miR-363-5p, miR-421-3p, miR-539-5p, miR-673-3p, miR-758-5p, are changed in the hippocampus when compared to GF mice (Chen et al., 2017). Interestingly, microbiota colonization of GF mice, restore changes of all miRNAs (Chen et al., 2017; Table 1). These miRNAs have reported to be related to neuropsychiatric diseases. Expression of miR-421-3p is changed in the amygdala and serum of rats exposed to acute traumatic stressors, suggesting its role in regulating posttraumatic stress disorder (Balakathiresan et al., 2014). Moreover, maternally separated mice significantly differ in the hippocampal miR-190a-5p expression levels (McKibben and Dwivedi, 2021).

In humans, administration of *Lactobacillus gasseri* CP2305 for 12 weeks improved stress-related behaviors in healthy young students who are with increased basal salivary cortisol and miR144 and miR144* expression levels (Nishida et al., 2017). Moreover, peripheral miR11/144* levels are elevated in medical students during the pre-examination period (Katsuura et al., 2012). Additionally, a decrease in miR144* levels during the post-examination period is correlated with decreased interferon-gamma (IFN-γ) levels such subjects (Katsuura et al., 2012).

Another aspect that hints at the microbiota relevance for miRNA expression is the commonly observed alterations in patients and animal models with irritable bowel syndrome (IBS). IBS is the most common functional digestive condition, characterized by recurrent abdominal pain and altered bowel movements (Saha, 2014). It is also considered as a complex and heterogeneous disorder with dysfunctional brain-gut axis and gut microbial dysbiosis (Saha, 2014). Not surprisingly, IBS presents high comorbidity with depression and anxiety disorders (Fond et al., 2014). Recent evidence shows increased miR-24 expression in the enterocytes (epithelial intestinal) in patients and a mouse model of IBS (Liao et al., 2016). By downregulating SERT expression, miR-24 inhibits serotonin reuptake transporter expression and aggravates IBS (Liao et al., 2016). Interestingly, miR-24 inhibitor alleviates intestinal pain and inflammation in IBS mice (Liao et al., 2016). Also, miR-16 and miR-103 are downregulated in the small intestine of IBS patients (Wohlfarth et al., 2017). miR-16 has been reported to mediate depression and anxiety (Song et al., 2015). These findings highlight the importance of new biomarkers involved in IBS and neuropsychiatric disorders, which could be new targets for disease treatment.

## The Role of MicroRNA on Depression, Anxiety, and Microbiota Composition

### The Potential Roles of MicroRNAs in Depression

Stress is one of the most studied etiologic factors for the onset of depression and other psychiatric disorders (Mandelli et al., 2015). Early life traumatic experiences, such as neglect and abuse, increase the risk of developing depression in adult life (Felitti et al., 2019). Prior experiences create a long-lasting change in central and peripheral systems associated with stress regulation, affecting how individuals adapt to future stressors (Ellis et al., 2006). Emerging data demonstrate that miRNAs activity could play a major role in long-lasting changes associated with depression pathogenesis and treatment (Baudry et al., 2010; Issler et al., 2014; Lopez et al., 2014).

Reduced *NOTCH1* gene expression, a transmembrane protein necessary for proper development, has been associated with depression and anxiety vulnerability in subjects exposed to traumatic early-life experiences (Steine et al., 2016). *NOTCH1* is a target for the miR-34 family. Blood analyses of 32 drug-naïve, first episode depressed patients display the increased levels of miR-34 alongside reduced *NOTCH1* mRNA levels, showing a negative association between the levels of miR-34 and *NOTCH1* mRNA (Sun et al., 2016). miRNA-9 is also suggested to mediate the adverse early life experience with future depression onset and severity, which could be linked with disturbed functional connectivity among prefrontal structures and subcortical limbic systems (He et al., 2021). miRNAs can be secreted into the extracellular fluid, acting as autocrine or paracrine communicators (Bayraktar et al., 2017), suggesting its essential role as potential biomarkers for stress-related neuronal dysfunction.

The relationship among stress, miRNA, and depression has also been validated in several animal models. Maternal deprivation can increase the hippocampal expression of miRNA Let-7a, which negatively correlates with reduced 5-HT_4_ receptors and anhedonia in the adult offspring (Bai et al., 2014). Chronic stress increases depressive phenotypes together with reduced miR-124 (Higuchi et al., 2016), increased miR-182 levels (Li et al., 2016) in the hippocampus, and increased miR-34a in the DRN (Lo Iacono et al., 2020). Chronic corticosterone administration can increase depressive phenotypes and miR-124 levels in the mPFC (Roy et al., 2017) and miR-34a levels in the hippocampus (Yi et al., 2020). Chronic stress-induced change in miRNA expressions in the mPFC are associated with altered gene expressions of BDNF, CREB and glutamate receptors, which are involved in synaptic transmission and plasticity (Dwivedi et al., 2015). Moreover, modulating miRNA expression by increasing miR-124 expression in the hippocampus (Higuchi et al., 2016), silencing miR-34 (Lo Iacono et al., 2020), and upregulating miR-135 (Issler et al., 2014) in the DRN, prevents stress-induced depression-like behaviors. Likewise, manipulating miRNA expression itself, such as upregulating hippocampal miR-182 by lentiviral overexpression, can induce depression-like behavior and increase the susceptibility to stress (Li et al., 2016). Therefore, accumulated evidence has proved that miRNAs could play a critical role as epigenetic regulators in depression pathogenesis.

MicroRNA expression profile is also sensitive to antidepressant treatment. Microarray analysis of blood from patients after a 12-week antidepressant drug treatment depicted an upregulation of 28 miRNAs and downregulation of two miRNAs when compared to the pre-treatment profile (Bocchio-Chiavetto et al., 2013). Chronic serotonergic antidepressant treatment downregulates serotonin transporter (SERT) and 5-HT_1A_ receptor, which are negatively modulated by the miR-135 (Issler et al., 2014). Congruently, depressed patients have reduced peripheral and central miRNA-135 levels, whereas peripheral levels are increased after 3 weeks of cognitive-behavioral therapy (Issler et al., 2014). *GRM4* which is targeted by miR-1202, can modulate serotoninergic and glutamatergic synaptic transmission. MiR-1202 is upregulated in the brain and downregulated in the blood of depressed patients, whereas both central and peripheral miRNA levels are normalized in patients taking antidepressant drugs (Lopez et al., 2014).

Selective serotonin reuptake inhibitors (SSRI), which compose most current first-line antidepressant treatments, have a delayed therapeutic onset of 4–8 weeks after treatment started (Trivedi et al., 2006). Chronic serotonergic antidepressant treatment reduces SERT protein expression without affecting its transcriptional level, suggesting translational-regulating mechanisms (Baudry et al., 2010). miR-1202 is reduced in the blood of depressed patients but can be normalized by chronic antidepressant treatment (Lopez et al., 2014). Accordingly, chronic but not acute serotonergic antidepressant treatment in neural progenitor cells upregulates expression levels of miR-1202 (Lopez et al., 2014). Likewise, chronic but not acute SSRI treatment in animals increases expression levels of miR-34a (Lo Iacono et al., 2021) and miR-16 (Baudry et al., 2010) in the DRN, whereas miR-34a is also increased in the hippocampus (Yi et al., 2020). It is suggested that such modulation of miRNA expression upon chronic but not acute serotonergic treatment is a key mediator of the antidepressant effects and one of the mechanisms associated with delayed therapeutic onset.

The relationship between miRNA and the serotonergic system has advanced the understanding of pathogenesis and the treatment of depressive disorders (Babicola et al., 2020). The miR-34 family is reported to be highly relevant to depression. Acute (Andolina et al., 2016) and chronic stress (Lo Iacono et al., 2020) upregulate miRNA-34a levels in the DRN. Knocking out the miR-34 family increases resilience to acute stress-induced anxiety (Andolina et al., 2016), chronic stress-induced depression (Lo Iacono et al., 2020), and switches the coping strategy toward active coping in the forced swim test (Andolina et al., 2018). Acute (Andolina et al., 2016) and chronic (Lo Iacono et al., 2020) stress increase serotonin levels in the mPFC, whereas miR-34 knockout prevents this increase and results in reduced expression of 5-HT_2C_ (Andolina et al., 2016), and increased corticotropin-releasing hormone receptor 1 (CRHR1) (Andolina et al., 2018) in the DRN. Therefore, increased miR-34 could modulate the DRN sensitivity to serotonin and corticotrophin-releasing hormone in response to stress exposure, resulting in increased serotonin input to the mPFC.

miRNAs could also function as crucial mediators of antidepressant treatments and potential therapeutic targets. Chronic corticosterone treatment upregulates hippocampal miR-34a levels associated with increased depressive phenotypes (Yi et al., 2020). Chronic treatments with SSRI or miR-34a antagonist counteract corticosterone-induced behavioral deficits in concurrent with restored hippocampal structural plasticity (Yi et al., 2020). Congruently, miRNA-16 infusion into the DRN counteracts chronic stress-induced depression to the same extent as chronic antidepressant treatment with fluoxetine (Baudry et al., 2010). Emerging studies have suggested the potential of manipulating miRNAs expression as a promising antidepressant treatment.

Investigating posttranscriptional mechanisms in depression increases our understanding of how individuals respond and adapt to stress. For example, rats displaying resilience to learned helplessness demonstrate decreased levels of some miRNAs compared to those susceptible to the stressor (Smalheiser et al., 2011). Of note, *CREB* mRNA, one of the most relevant proteins involved in plasticity and learning (Roy et al., 2013), is a target negatively modulated by many miRNAs (Smalheiser et al., 2011). Furthermore, such a mechanism shed light to unravel one of the most intriguing questions in the pharmacological antidepressant treatment, namely why SSRI acutely increases serotonergic transmission but takes several weeks to have therapeutic effect on symptom relief. As aforementioned, chronic but not acute antidepressant treatment can change the expression of miRNAs associated with key components of the serotonergic system that limit serotonin availability, for instance, SERT and 5-HT_2C_ receptors. miRNAs could be a potential therapeutic targets for tackling the etiology of depression with improved therapeutic response.

### The Potential Roles of MicroRNAs in Anxiety

Emerging evidence shows that dysregulation of miRNAs is involved in the stress response (Du et al., 2019), neurodegenerative diseases, and psychiatric disorders (Ha, 2011). miRNAs can be altered by stress, glucocorticoids, and mood stabilizers (Hunsberger et al., 2009), suggesting that miRNAs can also be involved in the pathophysiology of anxiety (Scott et al., 2015).

The majority of clinical studies have analyzed the expression of circulating miRNAs. Patients with generalized anxiety have upregulated peripheral levels of miR-633 and miR-4505, which correlates with the symptom severity (Chen et al., 2016). Panic disorder, on the other hand, has been associated with miR-22, miR-138-2, miR-148a, and miR-488 (Muiños-Gimeno et al., 2011). In animals, increased miR-34c levels in the amygdala are associated with acute and chronic stress-induced anxiety (Haramati et al., 2011). Moreover, Fisher 344 rats, a rat strain that displays higher anxiety levels, have increased expression of miR-18a and miR-124 (Uchida et al., 2008). miR-18a is known to inhibit the translation of glucocorticoid receptor (GR), whereas miR-124 reduces the GR expression levels (Uchida et al., 2008). Congruently, the expression levels of miR-18a are significantly correlated with the cortisol, corticotropin-releasing factor (CRF), and interleukin-6 (IL-6) plasma levels in humans (Wang et al., 2017a). miR-124-3p, on the other hand, is elevated, while GRs are decreased in the hippocampus of animals submitted to chronic corticosterone administration, an animal model used to mimic chronic stress (Wang et al., 2017b).

Studies also suggest the potential role of miRNAs in the treatment of anxiety. Chronic fluoxetine treatment elicits its antidepressant and anxiolytic effects in mice via increasing miR-16 levels in the serotonergic raphe nuclei, thus reducing the expression of serotonin transporter and increasing the bioavailability of serotonin in the synaptic cleft (Author et al., 2010). Therefore, miRNAs are involved in the pathogenesis of anxiety and depression are found to mediate anxiolytic drug treatments.

### Effects of MicroRNA on Microbiota Composition

The gut microbiota comprises approximately 10–100 trillion microorganisms, including 100–200 bacterial species and about 2–4 million genes (Ursell et al., 2012). Host genetic, diet, and diseases are key factors that can shape the microbiota composition of the host in the mammals (Thursby and Juge, 2017). Recent studies suggest the participation of miRNAs in modulating the gut microbiota composition (Singh et al., 2012). Some miRNAs can regulate bacterial gene transcription, affect bacterial growth and modulate the gut microbiota composition (Singh et al., 2012; Liu et al., 2016). Liu et al. (2016) have identified that miRNAs are abundant in mouse and human fecal samples and present within the extracellular vesicles in the gut lumen. Moreover, depletion of the DICER enzyme (miRNA-processing enzyme) in mice leads to exacerbated colitis and disturbed microbiota (Liu et al., 2016). On the other hand, when wild-type mice receive fecal miRNA transplantation from healthy mice, it restores fecal microbes and ameliorates colitis (i.e., inflammation of the colon), showing miRNAs influence gut health (Liu et al., 2016).

The gut microbiome has played a pivotal role in mediating the crosstalk between the gut and the brain. The gut-brain axis represents a critical communication system that, when disturbed, can lead to different immune, metabolic, and psychiatric disorders (Dinan and Cryan, 2017). Accumulated research has identified the importance of the gut-brain axis and various microbial-regulated molecular targets in the gut and the brain. Furthermore, it has been recently proposed that miRNAs are crucial signaling molecules to facilitate this bi-directional communication (Moloney et al., 2019).

MicroRNAs can have functional roles similar to hormones, influencing cellular function at a great distance from their original secretory sites (Bayraktar et al., 2017). miRNAs are a constitutive component of murine and human feces derived from host epithelium. They are detectable in feces and are essential for maintaining a normal gut microbiota (Liu et al., 2016). The microbiome regulates behaviors and physiology influenced by miRNAs (Foster et al., 2017). On the other hand, in germ-free (GF) mice, social interaction changes the expressions of miRNA in the amygdala, thus confirming the linkage between a functioning microbiome and sociability, suggesting that miRNA could influence behaviors modulated by the gut microbiome (Stilling et al., 2018). Another brain region influenced by miRNA is the hippocampus. A study has shown that inhibition of miR-124 in the mouse hippocampus improves performance in the Morris Water Maze task and a spontaneous alternation in the closed elevated plus-maze test (Malmevik et al., 2016), demonstrating the critical role of specific miRNAs on regulating behaviors associated with the hippocampus.

The gut microbiota can control gene expression in the brain through a miRNA network and targeted miRNAs (Chen et al., 2017). *In silico* analysis reveals that miR-294-5p targets the pathway associated with kynurenine metabolism and that genes related to this pathway are differentially expressed in GF mice devoid of all microbiota (Moloney et al., 2017). In the GF mice, it is found that miRNAs in the prefrontal cortex (PFC) and amygdala are sensitive to the presence of a gut microbiome. Upon recolonization, the expressions of some of these miRNAs are normalized. In the amygdala, miR-183-5p and miR-182-5p are decreased in GF mice, whereas expression is subsequently restored upon recolonization (Stilling et al., 2018). These miRNAs have been implicated in the amygdala response to fear and stress (Meerson et al., 2010). For example, miR-183-5p is increased in the circulation of depressed patients after antidepressant treatment (Bocchio-Chiavetto et al., 2013).

To date, there is evidence showing that a microbial product of *Bacteroides fragilis* lipopolysaccharide can act as a neurotoxin via induction of a series of miRNAs targeting genes that regulate synaptic plasticity, amyloidogenesis, and inflammatory signaling in the brain (Zhao et al., 2021). Also, other metabolites produced by the intestinal microbiota, such as tryptophan, butyrate, acetylcholine, norepinephrine, serotonin, dopamine can influence miRNA activity. They indirectly regulate astrocyte function and blood-brain-barrier integrity and even alter human behavior by disrupting normal neurotransmitter levels (Parker et al., 2020). Taken together, these data suggest that miRNAs are potentially involved in neuronal function and, hence, the pathologies of neurological disorders.

## Modulation of MicroRNA and Microbiota for the Treatment of Depression and Anxiety

Clinical studies have demonstrated the potential involvement of miRNA in modulating the antidepressant effect of probiotics in adults. A 4-week intervention with *Lactobacillus gasseri* probiotic improves depression symptoms, sleep quality, and bowel habits in adults with chronic stress and changes the miRNAs levels in the blood (Nishida et al., 2017). Moreover, as previously addressed, rodent studies further illustrate the role of microbiota in modulating miRNA expression and mood regulation. Microbiota depletion and recolonization bidirectionally influence the miRNA expression in the limbic system (Hoban et al., 2017). Probiotic administration, therefore, could be an effective treatment option.

A recent study examined how postnatal stress influences the affective behaviors of adolescent rodents and their microbiota composition (Karen et al., 2021). Daily maternal separation from postnatal day 5 to 10 induces anxiety- and depression-like behaviors and reduced gut microbiota diversity and richness (Karen et al., 2021). On the other hand, *Lactobacillus paracasei* supplementation alleviates anxiety-like behavior and normalizes stress-induced adrenocorticotropic hormone and corticosterone levels in rats (Karen et al., 2021). Comparisons between groups that have undergone maternal separation with and without administration of the probiotic showed that animals that received probiotics had a decrease in miR-132 and an increase in miR-124a levels (Karen et al., 2021). The potential antidepressant effects of probiotics are mediated by miRNA requires further investigation.

Emerging studies suggest that microbiota and microRNA modulation can be adjunct treatment strategies for depression and anxiety (Li et al., 2020). Physical exercise, which is known to have antidepressant and anxiolytic effects, can increase the number of beneficial microbial species (Mailing et al., 2019). A study has showed that 1 h of wheel running increases the relative abundance of *Lachnospiraceae*, a family of bacteria known to increase the synthesis of butyrate in the intestine, which is negatively correlated with anxiety-like behavior in adult C57Bl/6J mice (Kang et al., 2014). Butyrate is a short-chain fatty acid related to upregulated brain-derived neurotrophic factor (BDNF) expression in the rodent hippocampus and frontal cortex (Monda et al., 2017). Similar to exercise, butyrate also seems to increase neuroplasticity and has antidepressant effects by boosting serotonin levels (Monda et al., 2017). In addition, it has been reported that regular physical exercise can change the levels of miRNAs (Bye et al., 2013). Aerobic exercise increases the expression of miR-223 while reducing TLR4, MyD88, and NF-κB levels (Qu et al., 2020). Mice exposed to chronic stress have shown increased hippocampal levels of miR-223, which are reduced by 8 weeks of treadmill running (Qu et al., 2020). Moreover, 4 weeks of treadmill running increases the expression levels of miR-129-1-3p, miR-144-5p, and miR-10b-5p in the hippocampus (Fernandes et al., 2018). Eight weeks of antidepressant treatment leads to increased plasma levels of miR-144-5p in patients with depression or anxiety compared to their pre-treatment baseline levels (Wang et al., 2015). Taken together, changes in microbiota and, hence, miRNA expression can contribute to the antidepressant effect of physical exercise.

Diet is another factor that can modulate microbiota and miRNAs (Kang et al., 2014). Obese subjects have increased plasma levels of miR-155 (López et al., 2018), which are also increased in the plasma of depressed patients (Wang et al., 2018). Interestingly, miR-155 is related to the syntheses of pro-inflammatory cytokines, such as TNF-α, interleukin-6 (IL-6), and monocyte chemoattractant protein-1 (MCP1) (Migita et al., 2017). Mice fed with a high-fat diet decreases miR-137 levels in the cortex (Geekiyanage and Chan, 2011), whereas miR-137 deficiency leads to anxiety-like behavior and altered synaptic transmission and plasticity (Yan et al., 2019). Likewise, postmortem miR-137 levels are downregulated by 25% in the PFC of depressed patients with suicidal behavior (Smalheiser et al., 2012). High-fat diet-induces anhedonia-like behavior and decreases circulating leptin levels, which depend on gut microbiota composition (Hassan et al., 2020). Leptin is a hormone involved in regulating energy homeostasis and is associated with reduced depression and anxiety (Lu et al., 2006).

Taken together, these data suggest that the gut-miRNA crosstalk mediates antidepressant and anxiolytic effects and could as well be used as a target for new intervention strategies.

## Limitations and Future Perspectives

Emerging evidence has demonstrated the critical roles of miRNAs and the microbiota on psychiatric disorders, although the investigation of how they interact with each other on disease onset is still in infancy. miRNAs can mediate physiological responses in distal organs which are far apart from their production sites. Epigenetic factors have been proposed to modulate microbiota composition (Liu et al., 2016; Bayraktar et al., 2017). Notably, accumulated evidence from studies using GF mice has indicated that microbiota composition could influence miRNA expression patterns, suggesting direct interaction between gut microbiota and miRNA and their role in depression- and anxiety-like behavior (Chen et al., 2017; Hoban et al., 2017).

This interaction has been also supported by findings reporting the effects of antidepressant and anxiolytic treatments on regulating miRNA expression in the brain (Song et al., 2019) and microbiota composition (Lukić et al., 2019). Future studies identifying detailed mechanism underlying this interaction will help to understand more about the pathophysiology of depression/anxiety and pave the way for new therapeutic targets.

The development of new genetic animal models with knockout, knockdown, or overexpression of specific miRNAs will help to understand the importance of specific miRNAs and their targets on depression and anxiety. However, some miRNAs are present at chromosomes and necessary for embryonic development, making an experimental model of total miRNA depletion unfeasible (Hime et al., 2021). In addition, the multiplicity of targets for individual miRNA has created challenges in understanding the relationship between a single miRNA and a target protein (Liang and Li, 2007). However, up and downregulation of miRNAs in varied brain regions is a feasible alternative (Higuchi et al., 2016), whereas the combination of GF animals with structure-specific miRNA depletion could be alternative experimental approach.

Another alternative to this limitation is the use of non-mammalian models that extend our understanding of the molecular pathologies in human disease (Camkurt et al., 2017). Even though *Caenorhabditis elegans* and *Drosophila melanogaster* are evolutionarily distant from human physiology, and most pre-clinical drug tests are performed in rodents, these invertebrate model systems provide alternative approaches to conduct complementary research exploring common epigenetic mechanisms and biochemical processes that linked to neuropsychiatric pathologies (Camkurt et al., 2017).

**FIGURE 1:**
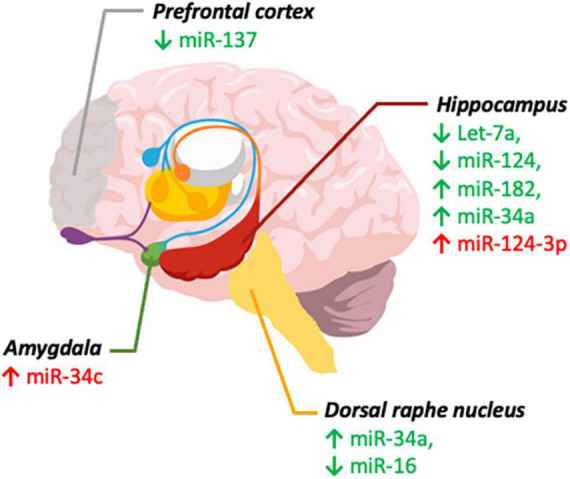
MicroRNAs implicated in depression and anxiety. miRNA levels in brain regions involved in depression (green) and anxiety (red). ↓ (Decreased levels) and ↑ (Increased levels). Created with Mind the Graph.

The clinical investigation of the crosstalk between miRNA and the gut microbiota, and their relationship with mental disorders have specific challenges. One question is to which extent changes of circulating miRNAs represent its changes in central miRNA levels (Jin et al., 2013). Moreover, studies would have to be well controlled because miRNA levels are affected by lifestyle factors, including diet, exercise, drug abuse, and medical conditions, imposing a significant challenge to form homogeneous groups (Jin et al., 2013). As summarized in Figure 1, psychiatric illnesses such as depression and anxiety are linked to altered miRNA expression in specific brain regions associated with mood regulation, such as the mPFC, hippocampus, DRN, and amygdala. Identifying these changes in circulation can help to develop new and more objective diagnostic tools for psychiatric disorders, as well as understanding the roles of aforementioned miRNAs in the relevant brain regions.

Understanding more about the interaction between miRNAs and the gut microbiota can deepen our knowledge in neuropathologies underlying depression and/or anxiety. The crosstalk between miRNAs and gut microbiota in psychopathologies could provide better understanding of the molecular pathways disrupted in these disorders and eventually pave the way for developing new therapeutic and diagnostic approaches.

## Conclusion

Accumulated studies have demonstrated that gut microbiota can influence miRNAs expression in different brain regions related to depression and anxiety, suggesting the potential role of specific miRNAs as an emerging treatment for depression and anxiety. The modulation of microbiota contributes to the mechanisms underlying antidepressant treatments, and comprehending the gut-brain axis leads to a more in-depth understanding of the neuropathology underlying depressive and anxiety disorders. Nonetheless, investigating the mechanisms through which the gut microbiota interacts with miRNAs in the brain is still in its infancy. Although probiotics, physical exercise, and diet intervention could contribute to future treatment strategies, further studies are needed to validate their therapeutic effects on clinical populations with depression, anxiety, and other psychiatric conditions.

## Author Contributions

JR wrote and edited the manuscript. DF, JY, and TL contributed to writing the manuscript. S-YY supervised and edited the manuscript. All authors approved the final version of the manuscript and agreed to be accountable for the content of the work.

## Conflict of Interest

The authors declare that the research was conducted in the absence of any commercial or financial relationships that could be construed as a potential conflict of interest.

## Publisher’s Note

All claims expressed in this article are solely those of the authors and do not necessarily represent those of their affiliated organizations, or those of the publisher, the editors and the reviewers. Any product that may be evaluated in this article, or claim that may be made by its manufacturer, is not guaranteed or endorsed by the publisher.
